# A novel promising Trichoderma harzianum strain for the production of a cellulolytic complex using sugarcane bagasse in natura

**DOI:** 10.1186/2193-1801-2-656

**Published:** 2013-12-06

**Authors:** Bruno Benoliel, Fernando Araripe Gonçalves Torres, Lidia Maria Pepe de Moraes

**Affiliations:** Centro de Biotecnologia Molecular, Departamento de Biologia Celular, Instituto de Ciências Biológicas, Universidade de Brasília, Brasília, DF, Brazil; Laboratório de Biologia Molecular, Departamento de Biologia Celular, Universidade de Brasília, Brasília, DF 70910-900 Brazil

**Keywords:** Sugarcane bagasse, Cellulases, *Trichoderma harzianum*, Brazilian Cerrado

## Abstract

Brazil is a major producer of agro-industrial residues, such as sugarcane bagasse, which could be used as raw material for microbial production of cellulases as an important strategy for the development of sustainable processes of second generation ethanol production. For this purpose, this work aimed at screening for glycosyl hydrolase activities of fungal strains isolated from the Brazilian Cerrado. Among 13 isolates, a *Trichoderma harzianum* strain (L04) was identified as a promising candidate for cellulase production when cultured on *in natura* sugarcane bagasse. Strain L04 revealed a well-balanced cellulolytic complex, presenting fast kinetic production of endoglucanases, exoglucanases and β-glucosidases, achieving 4,022, U.L^-1^ (72 h), 1,228 U.L^-1^ (120 h) and 1,968 U.L^-1^ (48 h) as the highest activities, respectively. About 60% glucose yields were obtained from sugarcane bagasse after 18 hours hydrolysis. This new strain represents a potential candidate for on-site enzyme production using sugarcane bagasse as carbon source.

## Introduction

Lignocellulosic residues derived from different agro-industrial activities represent a massive source of raw material for the production of fuels, chemical feedstock, foods and livestock feeds (Kumar et al. 2008). Brazil is a major producer of renewable feedstock including sugarcane which is essentially used for sugar and fuel ethanol production. In 2010, sugarcane production reached ~717.5 million tons (FAOSTAT 2012). A significant fraction of this biomass goes to industries for steam and electricity generation. The remaining fraction represents the ideal feedstock for the generation of high-value commodities as second-generation ethanol (Canilha et al. 2012).

The use of lignocellulosic biomass for the production of second generation ethanol requires a pretreatment for the liberation of carbohydrate polymers. A number of different strategies have been envisioned to convert the polysaccharides into fermentable sugars. One of them is accomplished by weak acid (chloridric or sulfuric acid) treatment (Betancur and Pereira Jr. 2010) to hydrolyze the hemicellulose fraction. The resulting solid fraction is then depolymerized by a chemical or enzymatic treatment. The later involves the use of different classes of hydrolytic enzymes generally produced by filamentous fungi. The conversion of cellulose to glucose involves the concerted action of three classes of enzymes: endo-β-1,4-glucanases (EC 3.2.1.4), exo-cellobiohydrolases (EC 3.2.1.91), and β-glucosidases (β-D-glucosidic glucohydrolases, EC 3.2.1.21). Hydrolytic enzymes represent a considerable cost in industrial biofuel plants. On-site enzyme production has been proposed as a way of lightening this burden. A fraction of lignocellulosic material partially hydrolyzed is diverted from the process and used as a cheap carbon source for enzyme production. Consequent reductions in enzyme storage time and downstream processing can thus lower the overall costs (Himmel et al. 1999; Tolan 2002).

The capacity of a particular microorganism to grow in lignocellulosic substrates is directly related to the production of a broad spectrum of enzymes that act synergistically to deconstruct the plant cell wall by depolymerization of substrates of different complexities (Andreaus et al. 2008; Kumar et al. 2008; Siqueira et al. 2010; Moreira et al. 2012). An assorted library of cellulolytic microbes should facilitate the development of optimal enzyme cocktails specific for locally available lignocellulosic biomass, such as sugarcane bagasse.

The Cerrado is the main savanna-like region in the Americas covering about 2 million km^2^. Although considered an important biodiversity hotspot (Myers et al. 2000) its microbial diversity has not been thoroughly assessed for biotechnological purposes. Our group has previously isolated an xylanase producing yeast from de Brazilian Cerrado (Parachin et al. 2009). The objective of this work was to evaluate the production of cellulases by a set of filamentous fungi isolated from the Brazilian Cerrado aiming their use as a potential source for on-site enzyme production.

## Materials and methods

### Microorganisms and fungal isolation

*Trichoderma reesei* Rut-C30 (ATCC 56765) (Montenecout and Eveleigh 1977) was used as reference strain. Decaying leaf litter encountered in the Cerrado’s soil from different areas around the city of Brasília was used as a source for fungal isolation. Collected samples were immersed in sterile distilled water and after vigorous agitation the suspension was subjected to serial dilutions and plated in a medium similar to the basic nutrient medium of Mandels and Weber (1969), with the exception that urea was omitted, a double amount of (NH_4_)_2_SO_4_ was included, and the peptone content was elevated by 20% (Szijártó et al. 2004), supplied with 1% carboxymethyl cellulose. Plates with standard inoculum of 10^3^ spores were incubated for 5 days at 30°C following Congo red staining (Ruegger and Tauk-Tornisielo 2004). Positive cellulolytic colonies were picked up and subcultured on potato dextrose agar (PDA - 0.4% potato, 2% dextrose, 0.5% peptone, 2% agar) slants and grown at 30°C for 10 days.

Submerged fermentations were performed in 500 mL Erlenmeyer flasks. Total of 10^6^ spores were inoculated into 100 mL of modified basic nutrient Mandels and Weber medium (Szijártó et al. 2004) supplied with 10 g.L^-1^ of different carbon sources. The cultures were incubated on a rotary shaker with an agitation rate of 200 rpm at 30°C for up to 120 hours. Throughout the cultivation, aliquots were withdrawn, centrifuged at 20,000 *g* for 5 min for cell and residual substrate analysis. Supernatants were stored at -20°C until the enzyme assays were carried out. Three biological replicates were performed for each condition.

### Enzyme and protein assays

Total cellulase, endoglucanase, and exoglucanase activities were determined using Whatman no. 1 filter paper (FPA), carboxymethyl cellulose (CMC, low viscosity), and microcrystalline cellulose (SIG) as substrates, respectively, according to standard conditions described by Ghose (1987). Reducing sugars, expressed as glucose liberated during reactions on FPA, CMC and SIG were quantified by the DNS method (Miller 1959). Endoxylanase activities were determined using xylan from oat spelts (XYL) as substrate according to Bailey et al. (1992). For all reactions one enzyme unit (U) was defined as the amount of biocatalyst that releases 1 μmol of the correspondent monosaccharide (xylose for xylanase and glucose for the other groups of enzymes) per minute under the assay conditions (30 min incubation at 50°C with 50 mM acetate buffer pH 5.0). β-Glucosidase activity was assayed in a 100 μL reaction mixture containing 3 mM ρ-nitophenyl-β-D-glucopyranoside (pNPG; Sigma, St. Louis, USA), 50 mM acetate buffer (pH 5.0), and an appropriate dilution of enzyme preparation. After 10 min incubation at 50°C, the reaction was stopped by adding 200 μL of 1 M Na_2_CO_3_, and ρ-nitrophenol (ρNP) release was monitored at *A*_405nm_. Enzyme unit was defined as the amount of biocatalyst that releases 1 μmol ρ-nitrophenol per minute under the assay conditions. Total extracellular protein content was measured using the Bio-Rad protein reagent according to the Bradford method (Bradford 1976) using bovine serum albumin (Sigma) as standard. All analyses were done in triplicate in a temperature-controlled incubator.

### Sugarcane bagasse hydrolysis

*In natura* sugarcane bagasse (SCB), used as substrate in the hydrolysis assays, was provided by Costa Pinto Mill (Piracicaba, SP, Brazil). This biomass was also subjected to pretreatments to generate partially delignified cellulignin (PDC). Acid and alkali pretreatments were carried out to increase the cellulose content in the materials by removing the hemicellulose fraction and partially removing the lignin fraction, respectively. The acid pretreatment consisted of incubating the solid material with a 3% (v/v) sulfuric acid solution (solid:liquid ratio of 1:4), while the alkali pretreatment was performed by incubating the material with a NaOH 4% (w/v) solution (solid:liquid ratio of 1:20). Both pretreatments were carried out at 121°C (1 atm) for 20 min.

Enzymatic hydrolysis was carried out using 1.0% partially delignified cellulignin (PDC) or *in natura* sugarcane bagasse (SCB) using a SCB-induced (72 h) culture supernatant. Suspensions were incubated at 50°C in 50 mM sodium citrate buffer (pH 5.0) for 18 h with regular sampling. Glucose concentration was determined usinga kit based on the glucose oxidase assay (Katal®) and total reducing sugar content was determined according to the method described by Miller (1959). The hydrolysis yield was determined using equation described by Maeda et al. (2011) from the carbohydrate contents of SCB and PDC previously determined by Castro et al. (2010a).

### Gel electrophoresis and zymogram analysis

SDS-PAGE analysis was performed to detect extracellular proteins. For visualization of carboxymethyl-cellulase and xylanase activities on gels a zymogram was performed according to Sun et al. (2008) with modifications. Briefly, proteins were separated on a 10% SDS-PAGE gel with either 0.15% CMC (low viscosity sodium salt) or 0.15% XYL (xylan from birch wood). The gel was washed twice in a solution of 0.5 M sodium acetate and 25% isopropanol at room temperatureto remove SDS. Proteins were renatured in 50 mM acetate buffer (pH 5.0) containing 5 mM β-mercaptoethanol by stirring the gel overnight at 4°C. The gel was then incubated in 50 mM acetate buffer (pH 5.0) at room temperature for 2 h followed by incubation at 50°C for another 2 h. The gel was stained in 0.2% Congo red for 1 h and distained with 1 M NaCl.

### Molecular identification

Standard protocols were followed for DNA manipulations (Sambrook and Russel 2001). Total fungal DNA was obtained from a mycelium grown on PDA as described by Roeder and Broda (1987). Ribosomal internal transcribed spacer (ITS) region was amplified from genomic DNA by PCR using primers ITS1 (5′-GCGGATCCGTAGGTGAACCTGCGG) and ITS4 (5′-GCGGATCCTCCGCTTATTGATATGC) (White et al. 1990). Double-stranded DNA sequencing was performed with the MegaBACE® Dye Terminator kit (GE Healthcare). Computer sequence analysis was carried out using the Phrap and Phred programs (Ewing et al. 1998).

Fungal isolates were identified via ITS sequence analysis using BLASTn search tools (http://www.ncbi.nlm.nih.gov). For taxonomic considerations, the obtained sequences were also used to include related species into phylogenetic trees. Sequence alignment was carried out using CLUSTALW (http://www.ebi.ac.uk/Tools/msa/clustalo/). Phylogenetic analysis was performed using MEGA v 4.0 software (Tamura et al. 2007). Bootstrap resampling analysis for 1000 replicates was performed to estimate the confidence of results. The DNA BarCode method for *Trichoderma* identification was carried out using the *Trich*OKEY v. 2 program (Druzhinina et al. 2005).

## Results

In a screening for cellulolytic activity a total of 13 isolates (L01-L13) from decaying leaf litter were selected after growth on cellulose as sole carbon source. Sequence analysis of PCR-amplified fungal ITS revealed a wide diversity of fungal taxa representing five genera: *Trichoderma* (5), *Penicillium* (4), *Aspergillus* (2), *Pestalotiopsis* (1) and *Curvularia* (1). All thirteen isolates demonstrated the ability to growth on cellulose as sole carbon source, but only four (L04, L08, L10, L11) showed a significant CMC hydrolysis halo when submitted to a rapid screening for cellulolytic activity on Congo red plate assay. The L04 isolate presented the fastest growth occupying all 9 cm diameter plate area in about 3 days while L10 took 4 days. Isolates L08 and L11 presented slower growth on CMC medium although the best colony/halo ratios, 0.72 and 0.63, respectively. No significant growth and/or activity hydrolysis halo on cellulosic substrate were observed in the other strain plates, therefore L04, L08, L10 and L11 isolates were selected for further studies.

To evaluate the cellulolytic productivity of the four selected isolates, submerged fermentation using CMC as sole carbon source was carried out for up to 120 hours. Every 24 hours, samples were withdrawn and assayed for cellulase activity. All analyzed isolates showed similar exoglucanase productivity when CMC was used as carbon source; the highest values observed were all around 1,200 U.L^-1^ in a culture time of 72 hours. L04 excelled at the endoglucanase productivity when compared to the others analyzed fungi, reaching the value of 2,206 U.L^-1^ (72 h). It represents about 6 times the production of L08, L10 and L11 at the same point of the growth curve. The production of β-glucosidase was also analyzed. The maximum activity detected for L04 was 2,938 U.L^-1^ (120 h), although at 96 hours of culture 2,350 U.L^-1^ has been achieved.

To evaluate the cellulolytic production ability of the selected strain in a complex substrate, SCB was used as carbon source in a submerged fermentation. Strain L04 demonstrated to be more efficient than the others analyzed strains when grown on SCB. The L04 maximum activities for endoglucanase, exoglucanase and β-glucosidase when grown on this substrate were 4,022 U.L^-1^ (72 h), 1,228 U.L^-1^ (120 h) and 1,968 U.L^-1^ (48 h), respectively. SCB seemed as a poor cellulase inductor for the other analyzed strains. No activities or very low endoglucanase and β-glucosidase activities were detected in the supernatant from L08, L10 and L11, and the maximum exoglucanase activity detected reached half of the values obtained by L04 on this substrate.

In terms of volumetric productivity, strain L04 reached the maximum values as early as 24 hours for β-glucosidase (52.0 U.L^-1^.h^-1^) and 48 hours for endoglucanase (64.2 U.L^-1^.h^-1^) when SCB was used as inducer (Table 1). When *T. reesei* Rut C30 was grown on SCB as carbon source, it presented maximum volumetric productivity values for endoglucanase, exoglucanase and β-glucosidase of 38.6 U.L^-1^.h^-1^ (72 h), 14.2 U.L^-1^.h^-1^ (24 h) and 29.3 U.L^-1^.h^-1^ (48 h), respectively. The *T. reesei* Rut C30 maximum detected activities for endoglucanase, exoglucanase and β-glucosidase when grown on this substrate were 3,795 U.L^-1^ (192 h), 567 U.L^-1^ (120 h) and 1,979 U.L^-1^ (192 h), respectively. Strain L04 was identified as *Trichoderma harzianum* (*Hypocrea lixii*) by phylogenetic analysis of its ITS1/2 regions and was selected for further analysis.

**Table 1:** **Maximum values of volumetric productivity observed for cellulases production by Cerrado isolates**

Strain	Substrate	Endoglucanase	Exoglucanase	β-glucosidase
L04	CMC	30.6 ± 1.2 (72)	25.9 ± 2.0 (24)	24.5 ± 1.0 (96)
	SCB	64.2 ± 6.4 (48)	10.2 ± 1.2 (120)	52.0 ± 0.4 (24)
L08	CMC	20.2 ± 1.2 (24)	24.9 ± 1.5 (48)	ND
	SCB	ND	6.3 ± 0.3 (96)	ND
L10	CMC	8.8 ± 0.3 (24)	30.9 ± 1.2 (24)	ND
	SCB	2.9 ± 0.1 (48)	36.8 ± 1.4 (24)	ND
L11	CMC	10.6 ± 0.7 (24)	25.2 ± 1.7 (48)	4.5 ± 0.2 (48)
	SCB	ND	12.2 ± 0.5 (24)	ND

Volumetric productivity (U. L^-1^ .h^-1^). Values in parentheses correspond to time of fermentation (h) when maximum results were observed. Not detected activity (ND).

The L04 enzymatic profile produced on SCB presented more xylanase, endoglucanase and β-glucosidase activities, even when grown on specific substrates (Table 2). However, partially delignified cellulignin (PDC) showed to be a poor inducer of L04 cellulolytic system (data not shown).

**Table 2:** **Maximum values of volumetric productivity observed for production of cellulases by** ***T. harzianum*** **L04 strain grown on different carbon sources**

Subtrate	Endoglucanase	Exoglucanase	Total cellulase	β-glucosidase	Xylanase
SCB	64.2 ± 6.4 (48)	10.2 ± 1.2 (120)	15.4 ± 0.2 (48)	52.0 ± 0.4 (24)	135.7 ± 1.7 (48)
CMC	30.6 ± 1.2 (72)	25.9 ± 2.0 (24)	3.8 ± 0.3 (24)	24.5 ± 1.0 (96)	44.5 ± 3.1 (48)
SIG	37.2 ± 1.6 (96)	3.1 ± 0.1 (24)	9.8 ± 0.6 (96)	40.4 ± 3.4 (48)	105.8 ± 5.3 (24)
XIL	19.0 ± 0.9 (24)	9.5 ± 0.6 (24)	3.8 ± 0.4 (48)	4.5 ± 0.3 (96)	132.5 ± 6.1 (24)

Volumetric Productivity (U. L^-1^ .h^-1^). Values in parentheses correspond to time of fermentation (h) when maximum results were observed.

**Figure 1:**
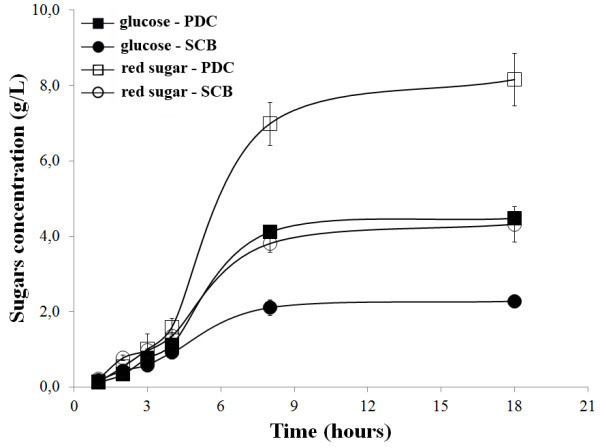
**Hydrolysis of 10% (w/v)** ***in natura*** **sugarcane bagasse (SCB) and partially delignified cellulignin (PDC) using enzyme cocktails produced by** ***T. harzianum*** **L04.** Concentrations of total reducing sugars and glucose during hydrolysis are indicated.

PDC and SCBwere subjected to enzymatic saccharification using the enzymatic cocktail produced by L04 grown on SCB. The enzyme/biomass loading ratio at this assay was equivalent to 5.0 FPU/g substrate. Figure 1 presents the temporal profile of the concentration of reducing sugars and glucose released by enzyme extract. After 18 h of saccharification, the L04 extract was able to release 4.32 g.L^-1^ of total reducing sugars from SCB and 8.16 g.L^-1^ from PDC. Glucose contents were also measured presenting values of 2.28 g.L^-1^ from SCB and 4.48 g.L^-1^ from PDC after 18 h of substrate hydrolysis. The released glucose concentrations were higher for PDC than SCB, however, in terms of glucose yield after 18 h of hydrolysis, calculated from the cellulose contents of SCB and PDC, the values were not different. The hydrolysis yield determined using SCB and PDC were 60.32% and 59.35%, respectively.

**Figure 2:**
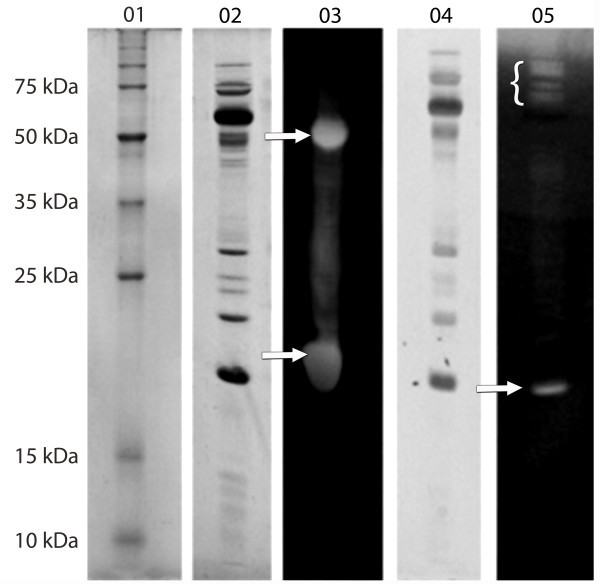
**Detection of cellulolytic and xylanolytic activities in** ***T. harzianum*** **L04 strain grown on SCB.** Molecular weights are indicated on lane 01. SDS-PAGE analysis (lanes 2 and 4). For detection of cellulolytic (03) and xylanolytic activities (05), cellulose and xylan were incorporated into the gel, respectively and stained with Congo red. The arrows in 03 indicate cellulolytic activities. The arrow and bracket in 05 the xylanolytic activities.

To detect cellulolytic and xylanolytic activities in L04 enzymatic cocktail used in the saccharification assay, electrophoretic analysis on SDS-PAGE and zymographic assays was also performed. The L04 protein profile presented two distinct molecular mass cellulolytic activities, ~50 kDa and 20 kDa (Figure 2, lane 3). Xylanolytic activities were also detected as a defined 20 kDa band and multiple activities over 75 kDa (Figure 2, lane 5).

## Discussion

In nature, the breakdown of plant materials is done primarily by fungi. In its natural environment saprophytic fungi can colonize the leaf litter and woody debris in humus or associated with plant matter in the soil. In order to grow on theses complexes substrates the ability to produce a broad spectrum of protein and polysaccharide hydrolyzing enzymes is required. For this reason, samples were collected from the Brazilian Cerrado environment and the fungi biodiversity analysed .

The wide diversity of fungal taxa found on Cerrado samples and the predominance of *Trichoderma* and *Penicillium* genera were expected, since the technique employed favors the isolation of the most common and abundant fungi (often referred as “generalists”) (Jeewon and Hyde 2007). The saprophytic condition of the Cerrado’s isolates reinforces their ability to use lignocellulose as carbon source, however not all of the isolated fungi were able to secrete significant amounts of cellulase activities for biotechnological use.

The strain L04 identified as *Trichoderma harzianum*, when grown on SCB presented the best ratios of cellulase production among all analyzed strains including *T. reesei* Rut C30. *T. harzianum* are frequently reported as control agent against fungal pathogens (Arantes and Saddler 2010; Banerjee et al. 2010). However, recent studies have also revealed the potential of this fungus for cellulase production and industrial applications (Ahmed et al. 2009; Castro et al. 2010a, 2010b). Likewise, *T. harzianum* has also become a promising system for xylanase production under appropriate conditions (Franco et al. 2004).

Castro et al. (2010b) had previously shown that *T. harzianum* IOC3844 exhibited an expressive production of endoglucanase activity with a fast kinetics with the exponential production phase detected between 31 and 72 hours of fermentation after a short lag phase. In this work, under the same assay conditions, strain L04 showed a shorter lag phase with an earlier exponential production phase starting before 24 hours when grown on SCB. Our results have shown that, unlike the other fungi analyzed in this work, L04 showed a shorter acclimation period when cultivated in a complex substrate such as SCB. This observation is particularly interesting when compared to *T. reesei* Rut C30, a widely used filamentous fungus strain used for the production of cellulolytic enzymes (Peterson and Nevalainen 2012). *T. reesei* Rut C30 shows a longer acclimation to lignocellulosic feedstock and is known to have a better performance for cellulase production when grown on pure cellulosic substrates then on lignocellulosic ones (Juhász et al. 2005), data confirmed by Castro et al. (2010c) where maximum values of volumetric productivity for *T. reesei* Rut C30 were obtained at 333 hours culture.

Strain L04 produced more xylanase, endoglucanase and β-glucosidase activities on different substrates irrespective of the carbon source (Table 2). The best results were obtained when L04 was grown on SCB. Partially delignified cellulignin (PDC) showed to be a poor inducer of L04 cellulases with delayed enzymatic production, contrary to expectations where delignification can improve enzyme production, since the large amount of lignin in SCB could irreversibly adsorb the enzymes produced during fungal cultivation. This would also be expected during hydrolysis where the absence of lignin probably reduces the adsorption of cellulolytic enzymes onto the lignin fraction of biomass (Berlin et al. 2005). This behavior was also reported for a *T. harzianum* strain isolated from the Amazon rainforest grown on pretreated sugarcane bagasse (Delabona et al. 2012).

The electrophoretic profile of L04 using SCB as substrate is similar to the one observed by Silva et al. (2012) using *T. harzianum* strain T4 cultivated in medium containing sugarcane bagasse. These authors showed by different electrophoretic techniques that *T. harzianum* was able to secret active multi enzymatic complexes with cellulolytic and xylanolytic activities which match the high molecular mass signal observed in L04 (Figure 2, lane 5). Both zymograms using CMC and xylan as substrate showed a signal around 20 kDa (Figure 2, lanes 3 and 5). The weaker signal in the xylan zymogram indicates the presence of a ~20 kDa xylanolytic activity which is compatible with other studies of this species that describe xylanase activities around this molecular mass with the lack of cellulase activity (Rezende et al. 2002; Lee et al. 2009; do Vale et al. 2012). The L04 cellulase activity around 20 kDa could correspond to the endoglucanase (EGIII) from *T. harzianum* IOC3844 characterized by Generoso et al. (2012) described with a low molecular mass, lack the cellulose binding domain (CBD) and able to degrade amorphous cellulose such as CMC.

In summary, L04 showed an interesting ability of producing significant yields of cellulase in a short culture time when grown on SCB. Also, it revealed a well-balanced cellulolytic complex, presenting fast kinetics for production of endoglucanases, exoglucanases and β-glucosidases. About 60% glucose yields were obtained from SCB and PDC after 18 hours of hydrolysis. We propose that *T. harzianum* L04 should be considered as a potential candidate for on-site enzyme production using *in natura* sugarcane bagasse as carbon source, in ready supply in a bioethanol production plant.
